# Determinants of Bacteriophage 933W Repressor DNA Binding Specificity

**DOI:** 10.1371/journal.pone.0034563

**Published:** 2012-04-03

**Authors:** Tammy J. Bullwinkle, Daniel Samorodnitsky, Rayna C. Rosati, Gerald B. Koudelka

**Affiliations:** Department of Biological Sciences, University at Buffalo, Buffalo, New York, United States of America; Niels Bohr Institute, Denmark

## Abstract

We reported previously that 933W repressor apparently does not cooperatively bind to adjacent sites on DNA and that the relative affinities of 933W repressor for its operators differ significantly from that of any other lambdoid bacteriophage. These findings indicate that the operational details of the lysis-lysogeny switch of bacteriophage 933W are unique among lambdoid bacteriophages. Since the functioning of the lysis-lysogeny switch in 933W bacteriophage uniquely and solely depends on the order of preference of 933W repressor for its operators, we examined the details of how 933W repressor recognizes its DNA sites. To identify the specificity determinants, we first created a molecular model of the 933W repressor-DNA complex and tested the predicted protein-DNA interactions. These results of these studies provide a picture of how 933W repressor recognizes its DNA sites. We also show that, opposite of what is normally observed for lambdoid phages, 933W operator sequences have evolved in such a way that the presence of the most commonly found base sequences at particular operator positions serves to decrease, rather than increase, the affinity of the protein for the site. This finding cautions against assuming that a consensus sequence derived from sequence analysis defines the optimal, highest affinity DNA binding site for a protein.

## Introduction

The major virulence factors in enterohemorrhagic *Escherichia coli* (EHEC) infections are Shiga toxins (Stx). In EHEC, the Stx encoding genes are carried on lambdoid prophages [1]–[6]. The *E. coli* O157:H7 strain EDL933 contains a lambdoid bacteriophage, 933W, whose genome includes the *stx*2 genes. The *stx2* genes are located within an operon controlled by the bacteriophage P_R_' promoter [7]. Transcription from this promoter ultimately depends on the activity of the 933W cI repressor protein, which directs the establishment and maintenance of the lysogenic state [8]. P_R_' is only active during lytic growth and therefore Shiga toxin is produced only during lytic, not lysogenic growth of the bacteriophage [9], [10].

The cI repressor protein controls expression of bacteriophage genes involved in regulating lambdoid bacteriophage development by binding to DNA sites in two operator regions O_L_ and O_R_. Each of these regions contain promoters that are activated or repressed by binding of the bacteriophage repressor protein to one or more of multiple, closely spaced binding sites. Efficient functioning of the genetic switch between lysis and lysogeny depends on repressor's ability to distinguish between the individual sites and to bind one site with the appropriate affinity relative to the other sites.

Our previous results showed that the relative binding affinities of 933W repressor for the individual sites in intact 993W O_R_ and 933W O_L_ differs from that observed in other lambdoid phages. For example, we showed that in intact 933W O_R_, 933W repressor does not bind its O_R_1 and O_R_2 sites at identical concentration [11], whereas in all other lambdoid phages, the repressors bind these two sites with nearly identical affinity. In these other lambdoid phages, simultaneous repressor occupancy of O_R_1 and O_R_2 is facilitated by cooperative interactions between two repressor dimers, one bound at each of these two sites. Our findings indicate that 933W repressor is apparently incapable of binding cooperatively to these two adjacent sites. Therefore 933W uses an alternative strategy for regulating its lysis-lysogeny decision. This strategy is based on the unique, differential affinity order 933W repressor displays for its naturally occurring binding sites [12].

**Figure 1:**
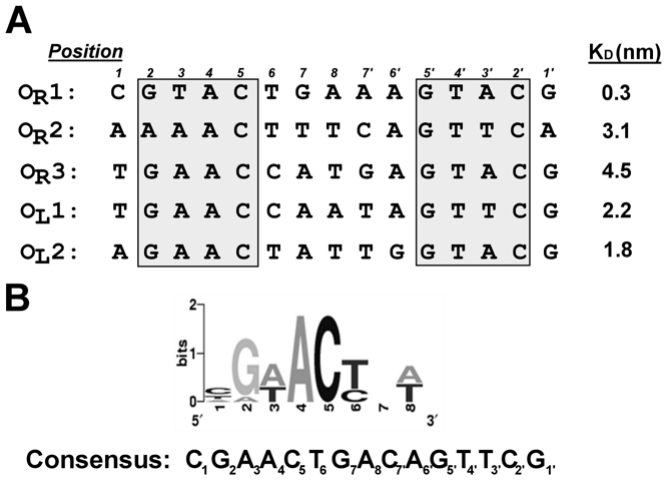
Naturally occurring 933W operator site sequences. *(A)* The sequences and affinity [12] (dissociation constants, K_D_) of 933W repressor binding for naturally occurring 933W binding sites. *(B)* Logo sequence alignment of the ten ‘half-sites’ [20] found within the five repressor operator sites and the consensus 933W repressor binding site sequence constructed from *in vitro* selection [11].

The base sequences at positions 2–5 and 2′–5′ in the five naturally occurring 933W repressor binding sites are incompletely conserved (Figure 1A). Using this data, we identified a consensus 933W repressor binding sequence [11]. This binding site is 15 basepairs long and its sequence is rotationally symmetric, with two half sites of sequence symmetrically arrayed about a central base pair (Figure 1B). By analogy with other bacteriophage repressors, we suggested that the DNA determinants for 933W repressor specific binding are located in the conserved region of the binding site sequence.

Since binding site discrimination by repressor is apparently crucial to formation of stable 933W bacteriophage lysogens, we wished to understand how 933W repressor recognizes its DNA sequence. To this end, we have used molecular modeling and binding studies to delineate the functional groups of each of the bases 933W repressor contacts. The results of these studies have provided insight into how DNA sequence ‘fine tunes’ the DNA binding affinity of 933W repressor. Also, comparison of the results presented here with those obtained with other lambdoid bacteriophage repressor proteins reveal a common sequence recognition motif that utilized by this class of proteins.

## Methods and Materials

### Bacterial Strains and DNA

All plasmids were propagated in JM101 [13]. 933W repressor was purified from the *E. coli* strain BL21(DE3)::pLysS (Novagen, Madison, WI) bearing a plasmid that directs its overexpression (p933WR) as described previously [11]. The S41A and K46A mutant 933WR repressors were constructed by site directed mutagenesis using p933WR as a template. The mutant proteins were purified as described [11].

**Figure 2:**
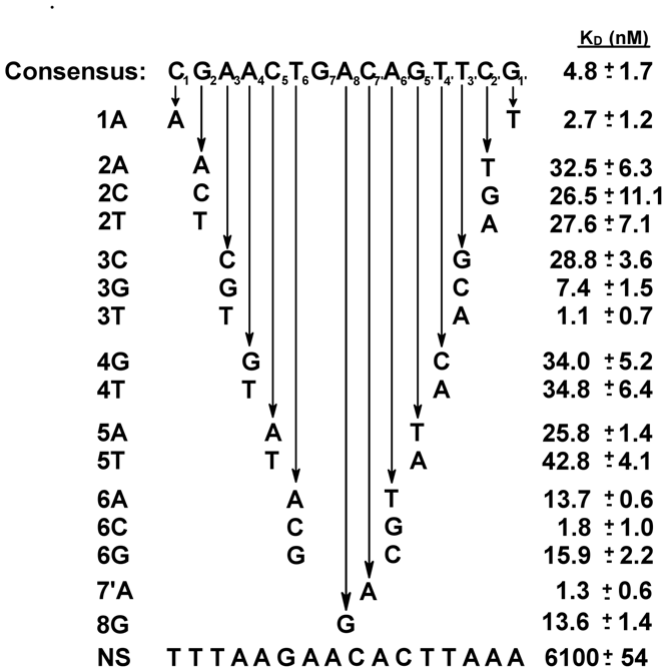
Affinities of wild-type 933W repressor protein for synthetic 933W operators. The affinities of 933W repressor for the substituted DNAs are expressed as the dissociation constants of these operators for 933W repressor. NS-DNA that does not contain a DNA sequence to which 933W repressor binds specifically. The affinity of 933W repressor for non-specific DNA is give as an intrinsic ‘per-site’ dissociation constant (K_D_^NS^ = K_D_^OBS^[2L-n], where L is DNA length and n is site size [28]). The protein concentrations used were corrected for activity.

To examine the effect of binding site mutations on the affinity of DNA for 933W repressor, we first designed a perfectly rotationally symmetric 15 base pair synthetic 933W repressor binding site sequence. This consensus sequence (see Figure 1B) is identical to the sequence described previously [11]. Complementary 60 base oligonucleotides encoding this 15 base site embedded within flanking DNA were obtained from Integrated DNA Technologies (Coralville, IA). We also obtained complementary 60 base oligonucleotides that bear symmetrically positioned mutations of the consensus sequence (Figure 2). Equivalent amounts of each pair of the complementary strands were mixed, heated to 85°C for 60 seconds and slow-cooled over four hours to anneal the two strands. Double-stranded DNA was separated from the individual single-strands by electrophoresis on 8% polyacrylamide gels in 1× TBE (89 mM Tris pH 8.9; 89 mM borate; 1 mM EDTA).

Naturally occurring binding site containing DNAs were generated by PCR from plasmids bearing 933W O_R_, constructed as described [11].

### Electrophoresis mobility shift assays

Two types of electrophoresis mobility shift assays (EMSA), a direct binding assay and a competition assay, were employed to measure the affinity of 933W repressor for its naturally occurring and synthetic DNA binding sites.

#### Direct binding

These experiments were performed as essentially described [11]. DNA containing the naturally occurring 933W repressor binding sites was obtained by either PCR using the desired templates and the standard forward and reverse M13 sequencing primers or by annealing oligonucleotides containing the 933W binding site sequence (see above). Following gel purification, the DNA fragments were radioactively labeled at their 5′ ends by incubating the DNA with γ-[^32^P]-ATP (6000 Ci/mmol) (Perkin-Elmer, Boston, MA) in the presence of T4 polynucleotide kinase (Epicentre, Inc. Madison, WI). O_R_1 DNA was also radiolabeled using α-[^32^P]-dATP (3000 Ci/mmol) (Perkin-Elmer, Boston, MA) in place of dATP in the PCR reaction to increase the radioactive signal and employ DNA concentrations below 0.1 nM. Labeled DNA was incubated with the specified concentrations of 933W repressor protein in binding buffer (10 mM Tris pH 8.0, 50 mM KCl, 1 mM MgCl_2_, 10% glycerol, 100 µg/ml BSA, 1 mM IPTG, 1 mM DTT) for 10 minutes at 25°C. The protein-DNA complexes were resolved on 5% polyacrylamide gels at 25°C. The electrophoresis buffer was 1× TBE. The amounts of protein-DNA complexes present on the dried gels were quantified using a Storm imager (GE Lifesciences, Piscataway, NJ). Values of the dissociation constant (K_D_) were determined by nonlinear squares fitting of the EMSA data to a hyperbolic equation using Prism 4.0 software (GraphPad Software Inc.). Each dissociation constant was determined from at least five replicate measurements.

#### Competition Assay

This method was used to measure the affinity of mutant consensus binding sites and a DNA molecule that did not contain a 933W repressor binding site. These experiments were performed as essentially described [14]. Briefly, following isolation from a polyacrylamide gel, the oligonucleotide encoding the 933W consensus binding site sequence was radioactively labeled at its 5′ ends as described above. A known amount of labeled consensus binding site DNA was mixed, in binding buffer, with an amount of 933W repressor that was previously shown to be sufficient to shift ∼50% of the labeled DNA binding site into a protein-DNA complex in the absence or presence of increasing amounts of unlabeled competitor binding site DNA. This mixture was incubated at 25°C for 10 minutes. The protein-DNA complexes were resolved, visualized and quantified as described above. Values of the dissociation constant (K_D_) were determined by nonlinear squares fitting of the competition data to the competition equation given in [14] using Prism 4.0 software (GraphPad Software Inc.). Each dissociation constant was determined from at least five replicate measurements. The dissociation constants (K_D_) obtained using the competitive binding and direct shift (where possible) assays are identical within experimental error.

### DNase I Footprinting

Annealed DNAs were radioactively labeled at the 5′ ends using T4 polynucleotide kinase as described above. To provide a uniquely labeled end, radiolabel from one strand of the DNA was removed by cleavage at an EcoRI site located near the 5′ end. Following phenol/chloroform/isoamyl alcohol extraction and ethanol precipitation, this DNA was incubated without or with 933W repressor in buffer (10 mM Tris pH 8.0, 50 mM NaCl, 1 mM MgCl_2_, 100 µg/ml BSA, 5 µg/ml chicken blood DNA, 1 mM DTT) for 10 minutes at 25°C prior to addition of sufficient DNase I to generate, on average, one cleavage per DNA molecule in 5 min of additional incubation. The cleavage reactions were terminated by precipitation with ethanol, dehydrated with sec-butanol and the DNA collected by centrifugation. The DNA was dissolved in 90% formamide solution containing tracking dyes. The products along with chemical sequencing reactions [15] derived from the same templates were resolved on 6% acrylamide gels containing 7M urea in 1× TBE. Cleavage products were visualized using a Storm imager (GE Lifesciences, Piscataway, NJ).

### 933W Repressor-Operator Complex Molecular Modeling

The first step toward generating a model of the interaction between the amino-terminal domain of the 933W repressor (933WR-NTD) and operator DNA was to generate a comparative model of the 933WR-NTD using Modeler [16]. In brief, the sequence of 933WR NTD (residues 1–75) was aligned against three template structures; the NTD's of lambda repressor (1LMB); P22 repressor (1ADR) and 434 repressor (2OR1) using ClustalW [17]. After obtaining the model-built structure, the peptide backbone atoms of residues 21–46 of the model-built 933WR-NTD structure were superimposed over the homologous amino acids in 434 repressor, residues 12–33, in complex with 434 O_R_1 DNA (2OR1, [18]) using SYBYL 8.1 (Tripos International, St. Louis, MO). These residues were chosen as they comprise the region of highest similarity between the two proteins as determined from a pairwise sequence alignment [17]. These residues correspond to the helix-turn-helix DNA binding structural motifs in these proteins. B-form DNA, bearing the sequence of the symmetric 933W operator, bases 1–8, was then superimposed over the DNA 434 repressor-operator structure, aligning the base 1 of the 933W binding site sequence with that of the −1 base of 434O_R_1. After removing the template protein and DNA structures, the resulting 933WR NTD-DNA complex was energy minimized using Amber_99 as implemented in SYBYL 8.1.

After convergence, the root mean square value of the resulting complex superimposed over the initial complex was 2.24 Å for all backbone atoms. To check the reliability of the final model, the minimization procedure was repeated several times. The final structures resulting from each round were highly similar and displayed a root mean square deviation of ≤0.5 Å.

## Results and Discussion

### DNA Sequence Determinants of 933W Repressor Affinity

The apparent inability of 933W repressor to bind cooperatively to adjacent sites and the absence of an O_L_3 site suggest that the operational details of the lysis-lysogeny switch in 933W differ from the established lambda paradigm. Our results indicate that the efficient functioning of the lysis-lysogeny switch in 933W bacteriophage depends on the unique order of preference 933W repressor has for its binding sites in O_R_ and O_L_ [12]. Hence the goal of this work is to examine how 933W repressor recognizes its DNA sites. The question of what constitutes a 933W repressor binding site is of interest since the identity of the bases at one or more positions in the strongest binding sites deviate from the derived consensus binding site sequence (Figure 1B, [11]).

Sequence analysis [19], [20] and our previous *in vitro* selection data [11] indicate that the 933W binding site is 15 bases long and consists of two rotationally symmetric blocks of sequence arrayed about a central base pair (Figure 1A). Within these blocks, the base sequences at positions 2–5 and 2′–5′ are both strongly conserved in the naturally occurring binding sites and are strongly preferred by 933W repressor in *in vitro* selection experiments. The base sequences at positions 6 and 7 and the central position are less well conserved and 933W repressor displayed less well-defined preferences for the base sequence at these positions [11]. Based on this analysis, we developed a consensus 933W repressor binding site sequence (Figure 1B).

### Effects base sequence on affinity of 933W repressor for DNA

To determine the identity of the bases within the 933W repressor binding site that affect its affinity for 933W repressor, we constructed a series of binding sites, bearing non-consensus bases at either symmetrical or all positions. The affinities of these sites for 933W repressor were measured by competition with the consensus binding site (Methods and Materials, [14], [21]) in an electrophoresis mobility shift assay. In a direct binding EMSA experiment, the mixture of protein and DNA is not at chemical equilibrium during the electrophoresis. If the dissociation of the complex is rapid relative to the rate of entry into the gel dissociation of the complex can lead to underestimates of the affinity of a protein for a given site [22]. However, our previous data indicate that affinities of 933W repressor for its naturally occurring operators and the consensus site determined from direct binding EMSA are identical, within error, to those determined by DNAse I footprinting [11], [12], a true equilibrium method [23]. This finding indicates that, as in many other cases [14], [21], [24]–[26], EMSA provides an efficient, accurate method for determining the affinity of 933W repressor for its binding sites [12]. Nonetheless, we were initially concerned that some of the 933W repressor-mutant DNA complexes we chose to examine may be much less stable than those we have previously studied. In this situation, direct binding EMSA may over-estimate the increase in K_D_ resulting from a particular deleterious base sequence change. Hence to ensure we accurately measured the affinity of 933W repressor for the mutant sites, we chose to perform these experiments using EMSA in competition mode. K_D_ measurements made by this method depend on the partitioning of the binding protein between a labeled DNA with known affinity and an unlabeled DNA whose affinity is being measured. Determination of K_D_ by this method is very accurate since all protein-DNA complexes experience the same conditions during gel entry and electrophoresis [27]. Hence the problems associated with determinations of K_D_ by multiple, separate direct binding EMSA experiments are minimized. Consistent with this assertion, we found that, where it was possible to measure, the values for the dissociation constants (K_D_) obtained using the competitive binding and direct shift EMSA are identical within experimental error. Hence, the dissociation constants determined by the competition EMSA accurately represent the affinity of the 933W repressor for the particular binding site.

As determined by both a direct binding and competition assay, the dissociation constant for 933W repressor binding to the consensus sequence is 4.8 nM. This value is similar to the affinity of this protein to its most of its naturally occurring binding sites, but 933W repressor binds this consensus site with ∼10-fold lower affinity than it binds to 933W O_R_1. This finding also indicates that the ‘best’ DNA binding site for 933W is *not* the site containing the consensus base at each position (see also below). The overall affinity of 933W repressor for this consensus sequence is similar to the affinities of other well-studied repressors (e.g., the repressors of λ, 434, P22) for their consensus and naturally occurring binding sites.

At 50 mM NaCl, the dissociation constant for binding of 933W repressor to DNA that does not contain a sequence resembling the 933W consensus sequence (i.e., ‘nonspecific’ DNA sequence) is 6.1 µM (assuming a site size of 18 base pairs determined by OH• footprinting). Therefore the specificity ratio (K_D_^NS^/K_D_^S^) for 933W repressor binding to O_R_1 is 2×10^4^, and for the consensus sequence is ∼1.3×10^3^. The affinity of a protein for its specific DNA site depends both on its sequence and solution conditions. In addition, the nonspecific DNA affinity of a protein sharply decreases with salt concentration. Hence it is difficult to compare specificity ratios between various proteins. When measured at 100–200 mM salt the specificity ratio [28] for other bacterial helix-turn-helix proteins varies from ∼10^3^ (gal repressor) [29] to ∼10^4^ (λ repressor, trp repressor) [30], [31] to as high as 10^7^ (lac repressor) [32]. Therefore, when the differences in salt concentration are accounted for, the specificity ratio of 933W repressor DNA binding is similar to that of other helix-turn-helix containing DNA binding proteins.

The sequence of the base at position 1 is poorly conserved among the naturally occurring 933W operators indicating it is of little importance in specifying a 933W repressor binding site. In *in vitro* selection experiments, 933W repressor also did not display a strong base preference at this position. Consistent with these observations, changing the base at position 1 from the consensus C•G pair to an A•T pair changes the DNA affinity of 933W repressor by less than 2-fold (Figure 2).

Except in one half-site of O_R_2, in the naturally occurring 933W operators, position 2 is occupied by a G•C base pair. Consistent with the strong sequence conservation at this position, changing the sequence at position 2 away from the consensus decreases the affinity of 933W for DNA by >6-fold (Figure 2).

**Figure 3:**
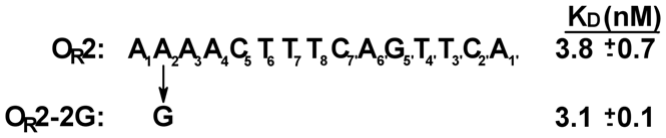
The effect of substituting position 2 base pair in 933W O_R_2 sequence on 933W repressor affinity. Affinities are expressed as the dissociation constants of these operators for 933W repressor. Dissociation constants were determined as described in Methods and Materials.

The strong deleterious effect of position 2 sequence changes on the affinity of binding site DNA for 933W repressor raises the question of how O_R_2, which contains a G•C base pair at position 2 in only one half-site, has such a relatively high affinity for 933W repressor. We tested the idea that the sequence context of the O_R_2 site may ‘blind’ the repressor from recognizing base substitutions at position 2 by changing the position 2 G•C base pair in one half site of O_R_2 to the consensus A•T sequence. Figure 3 shows that in the context of the O_R_2 sequence, this change has virtually no effect on the affinity of O_R_2 for 933W repressor. This contrasts with the >6 fold decrease in affinity of repressor for DNA when position 2 is changed in the context of the consensus sequence. Hence, operator sequence context affects 933W repressor's ability to recognize the identity of the base at position 2.

Changing the base pair at position 3 from the consensus A•T base sequence to C•G decreases the DNA affinity of 933W repressor by >6-fold. An A•T→G•C change at this position decreases the affinity of DNA for 933W repressor by ∼2-fold (Figure 2). 933W repressor binds to sites bearing a T•A base pair at position 3 with a >3-fold higher affinity than it does to sites bearing consensus A•T base pair (Figure 2). Therefore 933W repressor binds most tightly to sites bearing a non-consensus T•A base pair at position 3.

The base sequence of positions 4 and 5 in the naturally occurring operators is completely conserved, and the 933W repressor strongly prefers the consensus base sequences A•T and C•G respectively at these positions (Figure 1, [11]). Consistent with these observations, changing the identity of these base pairs away from the consensus decreases the DNA affinity of 933W repressor by >6-fold (Figure 2).

In 7 of the 10 symmetrically related half-sites of the naturally occurring 933W operators, position 6 is occupied by a T•A base pair and only C•G base pairs are found in the other three half-sites. Consistent with this pattern of base preferences, synthetic sites bearing either A•T or G•C pairs at this position bind 933W repressor much more poorly than do sites containing either T•A or C•G pairs. However, 933W repressor binds sites containing the lesser-preferred C•G pairs at positions 6 & 6′ with >2.5-fold higher affinity than it does to sites bearing the consensus T•A base pairs at this position. Thus, similar to what is seen at position 3, 933W repressor prefers sites bearing a nonconsensus base sequence at position 6.

Position 8, the base pair at the center of the 15 base pair operator, is occupied by an A•T (T•A) base pair. We wished to determine whether the identity of this base plays a role in determining the affinity of 933W repressor for its operator sites. Changing this base pair to G•C decreases the affinity of 933W for repressor ∼3-fold. Model building studies (below) suggest that the base at position 8, as well as positions 7 and 7′ are not closely approached by any amino acid side chains of 933W repressor. This finding suggests that 933W repressor recognizes the base sequence at these positions *via* indirect readout [33], [34]. To further explore how 933W repressor recognizes the bases at the center of the 933W binding site, we changed the base pair at position 7′ from C•G→A•T. An C•G→A•T change at position 7′ introduces a three base pair A-tract at the center of the 933W binding site. A-tracts located at the centers of P22 and 434 repressor binding sites, proteins which recognize the central base pairs of their respective operators by indirect readout [35], [36], increases the affinity of these proteins for their operators by facilitating collapse of the central minor groove [18], [33], [34], [37], [38].

Sequence analysis of naturally occurring 933W operators shows an apparent lack of sequence conservation at positions 7 and 7′. Also, 933W repressor does not demonstrate significant base preferences at positions 7 and 7′ in *in vitro* selection experiments [11]. Despite these observations, a C•G→A•T change at position 7′ *increases* the DNA affinity of 933W repressor by >4-fold. We speculate that introducing an A-tract at the center of the 933W repressor binding site affects 933W repressor's affinity for DNA *via* an indirect readout mechanism, i.e., altering the structure and/or flexibility of the unbound, similar to what is seen with other related DNA binding proteins [33]–[36].

As compared to what is observed with other protein-DNA complexes [39]–[41], changing the bases at symmetrically related positions of the 933W repressor binding site has a smaller effect on the DNA affinity of 933W repressor. We are uncertain why this is. It is known that site occupancy measured by EMSA can be affected by in-the-gel dissociation of the complex, meaning that this method could have underestimated K_D_. Thus, the effects of base sequence changes on affinity may even be smaller than we reported. If this were the case, that could suggest that 933W repressor discriminates between these various DNA sites solely by recognizing sequence-dependent differences in DNA structure [42], a mechanism known as indirect readout [35], [43]. Indeed we suggest that 933W repressor discriminates between operators bearing various sequences at positions 7, 8 and 7′ by indirect readout. However, our previous data and control experiments show that the affinities of 933W repressor for determined by EMSA accurately represent the affinity of 933W repressor for the various DNAs. Thus, while it possible that 933W repressor *only* uses an indirect readout mechanism in recognizing its operators, our control experiments and several lines of evidence given below indicate that 933W repressor uses a direct readout mechanism, i.e., direct amino acid base pair contacts, to discriminate between operators bearing base changes at positions 2–6 and 2′-6′.

### Model of the 933W repressor-DNA complex

**Figure 4:**
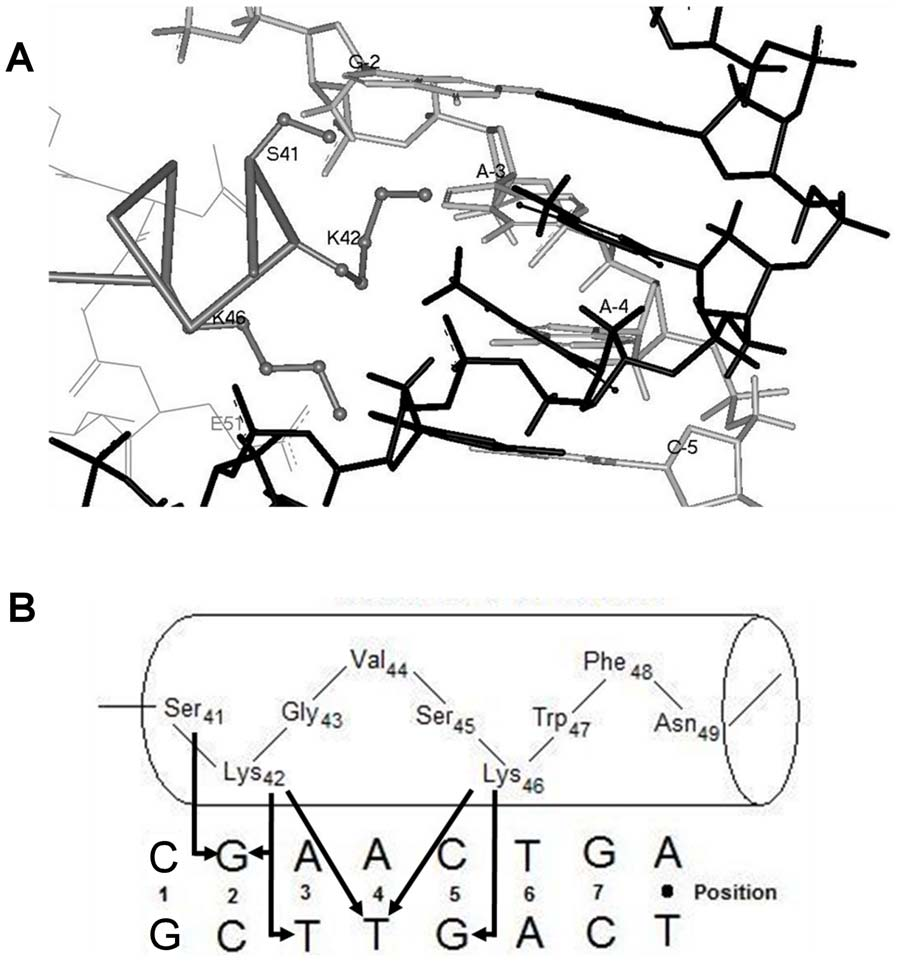
Model of the 933W repressor-operator complex. *(A)* 933W repressor in complex with the 933W consensus DNA site. The complex is oriented looking down the C-terminal portion of the DNA contacting (“recognition") to the N-terminal portions. The identity of the putative DNA contacting residues is indicated and highlighted in purple. The DNA is modeled on the right with the “top" strand (see Figure 1C) shown in grey and the complementary strand in black. *(B)* Schematic representation of the contacts identified from the model built structure between residues in the recognition helix of 933W repressor and bases in the 933W consensus half site DNA sequence. Predicted interactions are depicted by arrows.

In order to gain insight into the molecular determinants 933W repressor's base sequence preferences, we first created a three dimensional model of the complex between the amino terminal DNA binding domain (933WR-NTD) of 933W repressor and the consensus 933W binding site. As described in Method and Materials we constructed a model of the 933WR-NTD using the 434 repressor amino terminal domain (434R-NTD)-operator complex as a structural template. We used this protein-DNA complex as a template because of all structurally characterized helix-turn-helix-containing protein-DNA complexes, 434R- NTD is the closest sequence homologue to 933WR-NTD. The outcome of this model building effort is presented in Figure 4A. A schematic summarizing the proposed base-specifying contacts between residues in the recognition helix of 933W repressor and the DNA site is shown in Figure 4B.

The model structure suggests that the first residue (S41) of the “DNA recognition helix" interacts with the highly conserved guanine base of the G•C pair at operator position 2 (Figure 4). In the model, Nζ of K42 is positioned to form two hydrogen bond contacts, one with the O6 of the guanine base at position 2 and another with the O4 of the thymine of the conserved A•T base pair at operator position 3. The model indicates that 933W repressor does not make any direct hydrogen bonds with the base pair at position 4. However, the γ-CH_2_ group of K42 is within *van der Waals* contact distance of the C5-methyl on the thymine in the A•T base pair at position 4. In addition to K42, the C5-methyl on the thymine at position 4 is also apparently contacted by the ε-CH_2_ group of K46. At position 5, the Nζ of K42 is in a position to form a hydrogen bond contact with the O6 on the guanine residue of the C•G base pair at this operator position. Hence, the model apparently accounts for the DNA determinants of 933W repressor DNA binding specificity. The model suggests the 933W repressor's position 6 base preferences are specified by E51, a residue not in the ‘recognition helix’ (Figure 4A). Specifically, we find that the Oε2 of E51 contacts the N4 of the cytosine at position 6.

**Figure 5:**
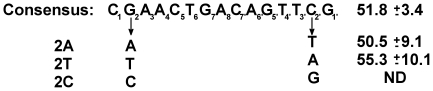
Affinities of S41A 933W repressor protein for synthetic 933W operators. Affinities are expressed as the dissociation constants of these operators for 933W repressor. The protein concentrations used were corrected for activity (ND = not determined).

To verify our proposed DNA binding specificity determinants of 933W repressor, we made substitutions at protein residues predicted to make primary contacts with DNA bases. The model suggests that changing S41 to an Ala should eliminate a base-specifying contact to position 2 and cause the mutant protein to have a different position 2 base preference than wild-type repressor. Consistent with this prediction, Figure 5 shows that 933WR S41A binds to sites containing either the consensus G•C or mutant T•A or A•T base pairs at position 2 with identical affinity. The lack of discrimination by the S41A mutant contrasts with the ≥6-fold lower affinity of the wild-type repressor for any site bearing a non-consensus base sequence at position 2. We note that the S41A mutant protein does not display any residual specificity for base pair 2. This finding contrasts with the apparent contact between K42 and bases at position 2, a contact that should cause the S41A mutant protein to maintain some position 2 base preferences. However, the observation that the S41A mutation decreases the overall affinity of 933W repressor to a greater degree (>10-fold) than does a position 2 base pair change (∼6-fold) suggests that this mutation has a pleiotropic effect on DNA binding specificity. Consistent with this idea, close inspection of the model built structure suggests that the precise positioning of the K42 side chain may be stabilized by an interaction with S41. Therefore we suggest that the S41A mutation destabilizes the contacts K42 makes with both positions 2 and 3, leading to the observed larger than expected decrease in overall specific DNA affinity of 933W repressor and complete loss of its position 2 base preferences.

According to our model, changing K46 to an alanine should eliminate two protein contacts per half-site and decrease the ability of 933W repressor to specifically bind to its cognate DNA site. Consistent with this expectation, the K46A mutant binds nonspecific DNA and the consensus sequence binding site with identical affinity (∼6.1 µM), indicating that this protein is completely incapable of specifically binding DNA. Hence the results of the protein mutational studies are consistent with the predictions of the DNA binding specificity determinants of 933W repressor.

**Figure 6:**
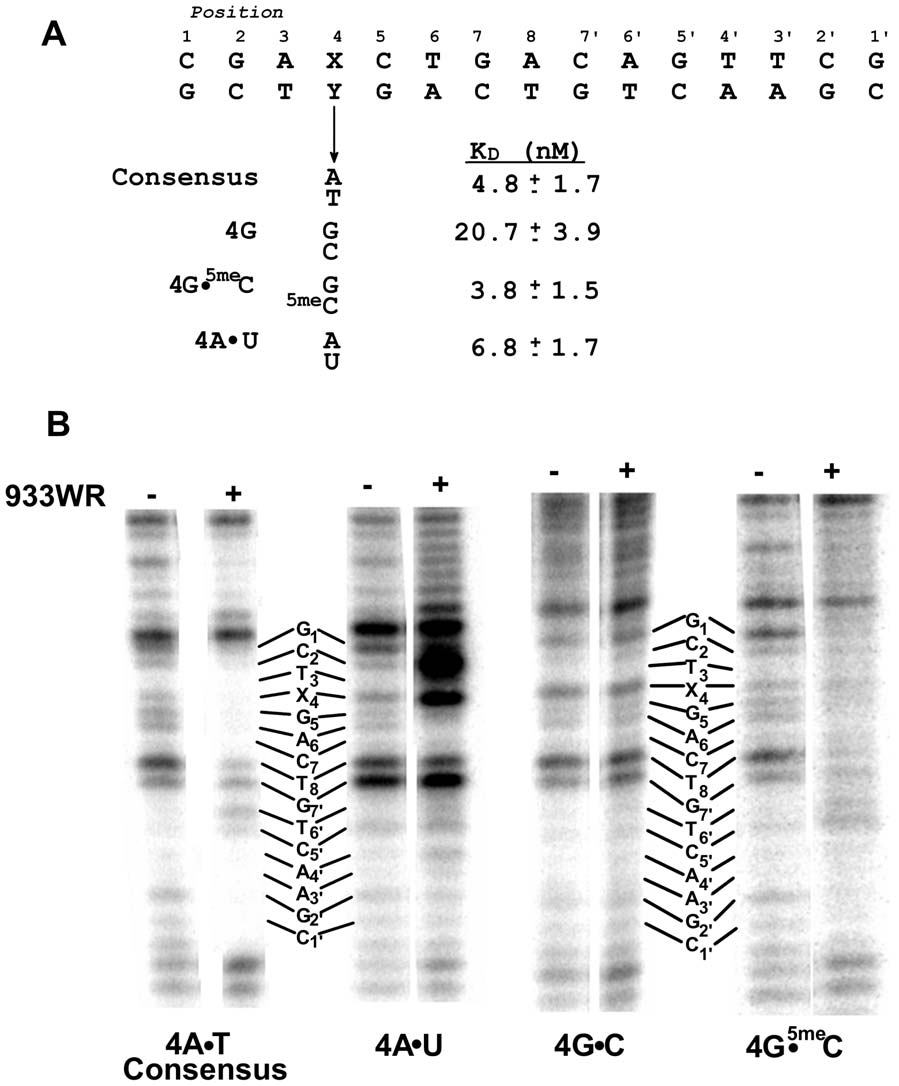
Role of Position 4 base substituents in determining 933W repressor affinity for DNA. (A) The base sequence at position 4 in one half site of the 933W binding site was changed as indicated. Dissociation constants were determined as described in Methods and Materials. (B) DNase I footprinting analysis of complexes between 933W repressor and 933W consensus sequence or its position 4 variants shown in (A) were performed as described in Methods and Materials. Shown is a Phosphoromager scan of the gel. The sequence of the operators are shown together with the positions of the bands resulting from DNase I cleavages in the absence (−) and presence (+) of saturating concentrations of 933W repressor.

Our model predicts that the interaction of K42 and K46 with the C5-CH_3_ on the thymine base at position 4 is a critical feature of 933W repressor's DNA recognition mechanism. We tested this prediction by examining the affinity of 933W repressor for DNA sites in which the A•T pair at position 4 in one half site is changed to G•C, G•^5me^C or A•U base pairs [44]. Consistent with the strong deleterious effect of an A•T→G•C substitution at positions 4 & 4′ on 933W repressor binding (Figure 6), the A•T→G•C change at just position 4 in just one half-site decreases the affinity of repressor for DNA by >5-fold. In contrast, 933W repressor binds the consensus site and a site bearing a G•^5me^C at position 4 with identical affinity. This result is consistent with the prediction that the C5-CH_3_ on the thymine base at position 4 is an important DNA binding specificity determinant of 933W repressor.

To further probe the role of the C5-CH_3_ on the thymine base at position 4, we also measured the affinity of 933W repressor for a DNA site bearing an A•T→A•U substitution at position 4. Based on the above results, we anticipated that, relative to the A•T containing DNA, removal of the C5-CH_3_ from the thymine at position 4 would significantly lower affinity of DNA for 933W repressor. However, the site bearing the A•T→A•U substitution binds 933W repressor with only slightly lower affinity than does the unsubstituted consensus DNA site (Figure 6A). This finding was surprising and appears to contradict the suggestion that the C5-CH_3_ plays an important role in mediating 933W repressor-DNA complex formation.

The precise role that the C5-CH_3_ group on the thymine at position 4 plays in mediating 933W repressor DNA binding is unclear from the results obtained with DNAs containing G•^5me^C and A•U at position 4. To help resolve this inconsistency, we probed the conformation of the unbound and repressor-bound DNAs using DNase I (Figure 6B). Although slight differences are observed near the site of substitution, the overall DNase I cleavage patterns of the unbound consensus and position 4 substituted G•^5me^C and A•U binding sites are highly similar. This observation suggests that the position 4 sequence changes do not significantly alter the unbound DNA conformation at or near the site of substitution.

Similar to our previously reported findings [11], 933W repressor bound to the consensus DNA site protects two ∼6 base pair regions of DNA from DNase I digestion (Figure 6B). The protected regions are symmetrically arrayed about the center of the rotationally symmetric binding site sequence and generally correspond to the positions of the conserved bases in the 933W repressor binding site. These protected bases encompass the region in which our model built complex structure indicates that 933W repressor directly contacts the DNA bases in the binding site.

The overall DNase I cleavage pattern of the 933W repressor-4G•^5me^C complex is very similar to that of the 933W repressor-consensus DNA complex. This finding indicates that the DNA conformation in the 933W repressor-DNA complex is not significantly affected by the 4G•^5me^C substitution. In the 933W repressor-4A•U complex, the DNA in the half-site containing the 4U substitution is extremely hypersensitive to DNase I cleavage and the half-site distal to the location of the 4U substitution is protected from DNaseI cleavage by 933W repressor. These findings indicate that 933W repressor contacts both half-sites of this DNA. Since 933W repressor is bound to the DNA, the observation of hypersensitive DNase I cleavage indicates the protein induces a DNA conformational change. Although we do not know the nature of this change, it likely results from protein-induced DNA bends or kinks [45], [46]. Therefore the DNase I cleavage analysis shows that near position 4 the conformation of the DNA in the 933W repressor-4A•U complex is significantly different from the 933W repressor-4G•^5me^C or 933W repressor-consensus DNA complexes. These findings indicate that removal of the C5-CH_3_ *via* uracil substitution at position 4 allows 933W repressor to induce this DNA conformational change.

The marked DNA conformational alteration in the 933W repressor-4A•U complex may explain why the position 933W repressor-4A•U complex has a similar stability to the 933W repressor-4G•^5me^C or 933W repressor-consensus DNA complexes. We suggest that this conformation change strengthens other 933W repressor-DNA contacts and thereby compensates for the loss of the C5-CH_3_ group in the 933W repressor-4A•U complex.

The base sequence at position 5 and 5′ is completely conserved in the naturally occurring 933W operators (Figure 1) and 933W repressor strongly prefers the consensus C•G base sequence at these positions [11]. Our model-built complex suggests that the position 5 and 5′ base sequence is specified by hydrogen bonded contact from the Nζ of K46 to the O6 on the guanine residue of the C•G base pair at these operator positions.

**Figure 7:**
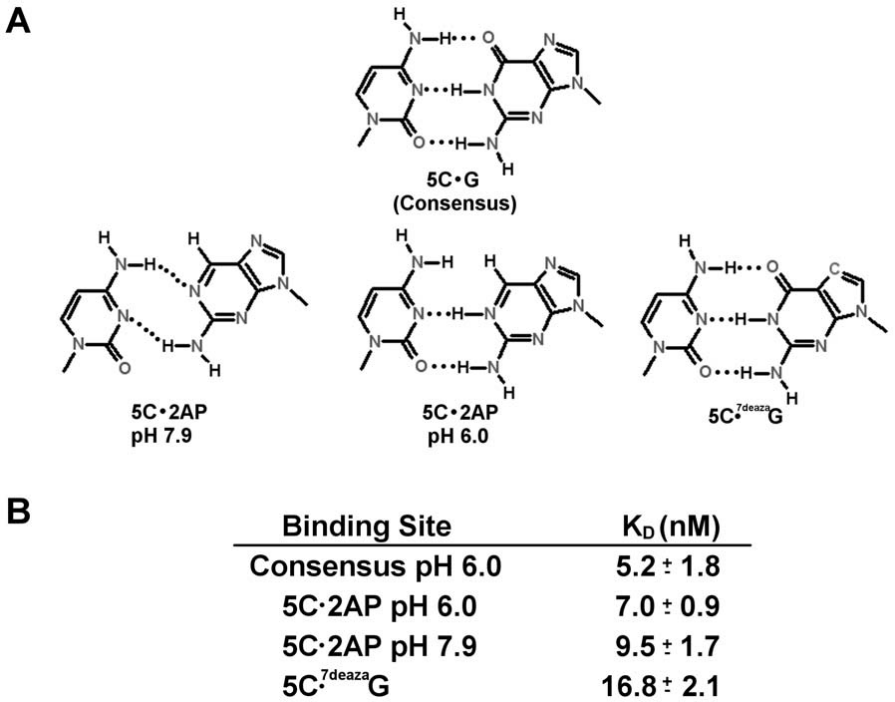
Role of Position 5 base substituents in determining 933W repressor affinity for DNA. The bases sequence at position 5 in one half site of the 933W binding site was changed as indicated. Dissociation constants were determined as described in Methods and Materials.

We tested this suggestion by examining the affinity of 933W repressor for operators in which the guanine base at position 5 is substituted with 2-amino purine (2AP) (Figure 7). This base is identical to guanine, except that it lacks the O6 atom. At pH 6.0, 2AP forms normal Watson-Crick base pair interactions with cytosine [47]. We found that at pH 6.0, substituting the position 5 C•G pair with a C•2AP pair decreases the DNA affinity of 933W repressor by less than 2-fold (Figure 7). We also found that at pH 7.9, where 2AP base forms a ‘wobble’ pair with cytosine [47], 933W repressor binds the DNA bearing a C•2AP at position 5 with a only 2-fold lower affinity than it binds the unsubstituted consensus DNA site (Figure 7). These observations indicate that the O6 on the guanine residue at position 5 contributes to, but is not a major specificity determinant for, 933W repressor DNA binding.

Analysis of the model of the 933WR-NTD-DNA complex shows that a rotation of the K46 side chain would allow the Nζ of this residue to contact the N7 of the guanine base at position 5 and 5′ instead of the O6 atom. To test whether the N7 atom of the guanine at position 5 is contacted by 933W repressor, we substituted this base with 7-deaza-guanine (^C7^G) and determined the affinity of 933W repressor for DNA sites bearing this substitution (Figure 7). The G→^C7^G change at position 5 decreases the affinity of 933W repressor for DNA by >5-fold relative to its affinity for the unsubstituted consensus binding site sequence. The decreased affinity of 933W repressor for the substituted site is consistent with the idea that the N7 of the guanine residue at position 5 is a specificity determinant for 933W repressor DNA binding.

Our results demonstrate that our 933WR NTD-DNA complex model structure provides an accurate framework for understanding several aspects of 933W repressor's operator recognition mechanism. As a consequence, we can use this model to help explain 933W repressor's relatively promiscuous base position 3 base preferences. 933W repressor displays a hierarchy of affinities for operators bearing substitutions at position 3, T•A>A•T>G•C>>C•G. The model complex shows that in the consensus binding site, the Nζ of K42 contacts both the O6 of guanine at position 2 and the O4 of the thymine at position 3. In energy minimized complexes with various sequences at operator position 3, K42-Nζ maintains contacts with C = O groups at both positions 2 and 3, *except* when position 3 is a C•G pair. In the 3C operator, sequence-dependent changes in DNA conformation and concomitant movement of the K42 side chain, causes this residue to lose contact with the guanine O6 at position 2. Consequently in the 3C complex, this residue only contacts the guanine O6 at position 3. We suggest that this loss of position 2 contact as a consequence position 3 C•G substitution causes the strong decrease in the affinity of 933W for the 3C binding site.

Inspection of the model 933WR-NTD complex does not provide a ready rationalization for why a G•C→A•T substitution at position 2 in the context of the consensus binding site decreases 933W repressor's affinity for DNA (Figure 2), but the identical change made in the context of the O_R_2 sequence has no effect on 933W repressor affinity (Figure 3). Model building suggests that this change would eliminate the contacts made by K42 with the base at position 2, and should thereby decrease the affinity of 933W repressor for DNA.

How could the O_R_2 DNA sequence context ‘blind’ 933W repressor to a position 2 G•C→A•T base change? Examination of the O_R_2 sequence reveals that the A•T base pair at position 2 is part of a four base pair long A-tract sequence. Sequences containing >3 ^5′^ApT^3′^ and/or ^5′^ApA^3′^ steps adopt the B′ state [48]–[50]. This state is conformationally distinct from that assumed by mixed sequence DNA. Among other features, A-tract DNA has a narrow minor groove [48], [51], displays negative propeller twisting of the base pairs [52], [53] and has a propensity to bend DNA when appropriately phased [54]–[56]. We have already shown that the stability of 933W repressor-DNA complexes can be remarkably influenced by changes in DNA conformation. Therefore we suggest that in 933W O_R_2, the unusual structure of A-tracts either alters the strength of other 933W repressor-DNA contacts, compensating for the loss of K42 contacts to the base at position 2 or allows an alternative contact between protein & DNA at this position. The B′-state is not supported by G/C containing sequences hence the region surrounding position 2 in O_R_2-2G would not be anticipated to assume this conformation [57]. We suggest that the loss of A-tract ‘stabilization’ in the O_R_2-2G operator-repressor complex is compensated by the reestablishment of ‘consensus-like’ contacts between K42 and the guanine base at position 2. This compensation could allow 933W repressor to bind O_R_2 and O_R_2-2G sites with similar affinities.

The idea that the DNA contacts made by K42 may depend on sequence context and the observation that the absence of a C5-CH_3_ group on the pyrimidine at position 4′ affects the DNase I protection pattern of 933W repressor-DNA complexes only in the half-site bearing the mutation (Figure 6B) suggests that the interactions made by each monomer of the DNA bound 933W repressor dimer are not required to be identical in each half site. As observed with both bacterial and eukaryotic DNA binding proteins, sequence dependent alterations in DNA contacts can lead to changes in the structure and function of the bound protein [58]–[63]. Therefore as has been observed for bacteriophage 434 repressor [60], [61], it is possible that sequence differences between the naturally occurring 933W operators could have affects on 933W repressor beyond simply modulating its affinity for DNA.

As demonstrated in Figure 6, the thymine C5-CH_3_ group is the sole determinant of 933W repressor protein's position 4 base sequence preferences. The utilization of thymine methyl groups as sole specificity determinants of inner operator positions is a common feature of DNA recognition by all well-studied helix-turn-helix containing bacteriophage repressor proteins [18], [33], [64], [65]. Similar to 933W repressor-DNA complexes, in all these cases, the thymine C5-CH_3_ group fits into a pocket formed by the aliphatic atoms of amino acid side chains in the recognition helix. The functional groups of these residues contact DNA bases at other positions in the binding site. Neither the closely related LexA repressor, nor other well-studied bacterial HTH-variant proteins (e.g. cAMP receptor protein or its relatives), nor the structurally similar homeodomain proteins in eukaryotes, share this feature in their DNA recognition mechanism. The juxtaposition of the DNA recognizing residues with DNA in all these protein-DNA complexes differ significantly from those of the phage repressors. Hence, this form of base specifying contact appears characteristic of the phage repressor class of DNA binding proteins.

In all lambdoid bacteriophages, establishment and maintenance of lysogeny, as well efficient induction of the lysogenic phage, requires that the bacteriophage repressor bind with appropriate affinity to each of the individual sites within O_R_ and O_L_. For example, tight repressor binding at O_R_1 and O_R_2 is needed, respectively, to repress transcription from P_R_, the promoter needed for expression of lytic genes, and activate transcription from P_RM_, the promoter responsible for driving repressor expression in a lysogen. In all well-studied lambdoid phages, the intrinsic affinity of repressor for its cognate O_R_1 is much higher that for its cognate O_R_2 and the repressor's intrinsic affinity for O_R_3 is typically higher than that for O_R_2. Nonetheless in these other phages, cooperative interaction between two repressor dimers allows the repressor to bind 1) O_R_1 and O_R_2 at identical concentration and 2) to bind both of these sites at a significantly higher affinity than to O_R_3. However, the bacteriophage 933W repressor is apparently incapable of cooperative DNA binding. [11], [12]. The relative affinity of 933W repressor for its sites in O_R_ (Figure 1) are qualitatively and quantitatively different than what is seen with other well-studied lambdoid bacteriophage repressors, e.g., λ, 434 and P22 [30], [41], [66]–[70]. Numerical simulations that assumed that 933W repressor does not cooperatively bind DNA and using the intrinsic affinities determined *in vitro* on linear DNA substrates accurately predicted 933W repressor's gene observed gene regulatory activity *in vivo* [12]. This observation argues that the operator binding affinities of 933W repressor *in vivo* are identical to those determined *in vitro*, unaffected by DNA supercoiling or putative supercoiling-facilitated cooperative interactions.

As a consequence of its inability to bind DNA cooperatively, at any given 933W repressor concentration, its occupancy of any particular DNA binding site depends solely on its intrinsic affinity for the site (Figure 1), which in turn depends on how well the sequence of a given binding site matches that preferred by 933W repressor. How then do the sequences of 933W operators ‘fine tune’ their affinity for 933W repressor? We noted earlier [11] that the sequence of O_R_1, the site we subsequently showed is the highest affinity naturally occurring binding site [12] (see also, Figure 1), differs markedly from the derived consensus sequence, specifically at positions 3, 7′ and 3′. Usually the consensus base sequence derived from analysis of base frequencies at each position in a protein's DNA binding site is the base sequence that supports high affinity binding. We show here that at several positions, the base sequences that *deviate* from consensus in high affinity sites are in fact the *preferred* base sequences.

If these base sequence differences between O_R_1 and the consensus are the sole cause of the difference in affinity of these two DNAs for 933W repressor, the sum of ΔΔG for each change should account for the observed difference in affinity. The free energy for 933W repressor binding O_R_1 is −12.93 kcal/mol and that for binding to the consensus sequence is −11.3 kcal/mol, a ΔΔG of −1.63 kcal/mol. In the context of the consensus sequence, change in free energy (ΔΔG) for the symmetrical position 3/3′ A•T→T•A change is −0.87 kcal/mol, and that for the 7′ C•G→A•T change is −0.77 kcal/mol. The sum of these free energy differences is −1.64 kcal/mol, which almost precisely matches the ΔΔG between O_R_1 and the consensus site. This finding argues 1) that the sequence differences between the consensus and O_R_1 at positions 3, 3′ and 7′ completely account for their differences in affinity of these DNAs for 933W repressor and 2) supports the idea that the ‘fine tuning’ of 933W repressor binding to its naturally occurring sites depends on how well the sequence of a given binding site matches the base sequence preferred by 933W repressor, not the consensus base sequence. While our data do not allow us to perform a complete analysis of the effect of all base sequence differences between the consensus site and the other naturally occurring sites, our findings do qualitatively support the suggestion that the consensus sequence does not define the preferred sequence. For example, the second ‘strongest’ naturally occurring site, O_L_2, which also binds 933W repressor more tightly than consensus, contains favored, but non-consensus base pairs at position 3′ and 6′ as does the similar strength O_L_1 site.

Together our observations indicate that 933W operator sequences have evolved in such a way that the presence of the most commonly found base sequences at particular positions serve to decrease, rather than increase, the affinity of the protein for the site. This finding serves to caution against assuming that a consensus sequence derived from sequence analysis defines the optimal, highest affinity DNA binding site for a protein.
